# Ultrasonographic Appearance of Acute Prostatitis and Prostatic Abscesses in Dogs

**DOI:** 10.3390/ani16182827

**Published:** 2026-09-08

**Authors:** Stefano Spada, Gaia Pagani, Johannes Herbel, Bianca Lourdes Frehner, Joana Rodrigues Carvalho, Hélène Jainek, Martina Gavezzoli, Andrea Major, Sebastian Patrick Arlt

**Affiliations:** 1 Clinic of Reproductive Medicine, Vetsuisse Faculty of Zurich, University of Zurich, 8057 Zurich, Switzerland; stefano.spada@uzh.ch (S.S.); gaia.pagani@uzh.ch (G.P.); johannes.herbel@uzh.ch (J.H.); biancalourdes.frehner@uzh.ch (B.L.F.); joana.rodriguescarvalho@uzh.ch (J.R.C.); helene.jainek@uzh.ch (H.J.); 2 Department of Veterinary Science, University of Parma, 43126 Parma, Italy; martina.gavezzoli@unipr.it; 3 Clinic for Small Animal Internal Medicine, Vetsuisse Faculty, University of Zurich, 8057 Zurich, Switzerland; amajor@vetclinics.uzh.ch

**Keywords:** prostatitis, prostatic abscess, ultrasound, dog

## Abstract

Prostatitis is one of the most common prostatic disorders in dogs, although its ultrasonographic features remain poorly characterized. The aim of this retrospective study was to describe the ultrasonographic findings of canine prostatitis and prostatic abscessation and to investigate their association with clinical and laboratory abnormalities. Medical records of 35 client-owned dogs with cytologically or histologically confirmed acute prostatitis were reviewed. Clinical, ultrasonographic, laboratory, bacteriological, treatment, and follow-up data were collected. The most common ultrasonographic findings were inhomogeneous parenchymal echotexture, intraparenchymal cystic lesions, capsular edema, periprostatic steatitis, and asymmetry of the prostatic lobes. Prostatic abscesses were detected in 25 dogs and showed marked variability in size and morphology. Abscess diameter was positively correlated with body weight (*p* = 0.001; rho = 0.6) and negatively correlated with the percentage of neutrophils with toxic granulation (*p* = 0.02; rho = −0.4). Most dogs recovered following medical or combined medical–surgical treatment. Follow-up examinations demonstrated resolution of the inflammatory changes surrounding the prostate, whereas parenchymal inhomogeneous echotexture frequently persisted. These findings provide a comprehensive ultrasonographic description of canine prostatitis and support the use of ultrasonography as a valuable tool for diagnosis and follow-up.

## Simple Summary

Prostatitis is one of the most common diseases affecting the prostate gland in dogs. Although ultrasonography is routinely used to evaluate the canine prostate, the sonographic features of prostatitis have been poorly reported. The aim of this retrospective study was to describe the ultrasonographic features of 35 dogs diagnosed with acute prostatitis with or without prostatic abscesses. Moreover, we aimed to investigate potential correlations/associations with clinical and laboratory findings. The most common ultrasonographic abnormalities were prostatic enlargement, lobe asymmetry and an inhomogeneous parenchyma. Additional findings included cystic lesions, capsular swelling of the prostate, inflammation of the periprostatic fat tissue, reactive iliac lymph nodes and prostatic abscesses. Prostatic abscess size was negatively correlated with the percentage of neutrophiles with toxic granulation. Most dogs recovered after medical or combined medical–surgical treatment. Follow-up ultrasound examinations showed resolution of the inflammatory changes surrounding the prostate, whereas structural alterations within the gland persisted. The results provide a detailed description of the ultrasound appearance of canine prostatitis and suggest that ultrasound may be useful not only for diagnosis but also for monitoring treatment response. Further prospective studies are needed to determine the prognostic value of specific ultrasound findings.

## 1. Introduction

The prostate is the only relevant sex gland in dogs, and its main function is to provide prostatic fluid, which is important for semen motility and viability, directly impacting male fertility. Benign prostatic hyperplasia (BPH), prostatitis and prostatic neoplasia represent the most common prostatic diseases in dogs [1,2]. Among these conditions, prostatitis is considered the second most frequent disorder and has been described to account for 20–40% of all canine prostatic diseases [3,4]. Prostatitis consists of an acute or chronic inflammation of the prostate gland, most associated with bacterial infection, particularly involving *Enterobacteriaceae* spp. [5,6].

Inflammatory changes may coexist and occur as a consequence of other prostatic disorders, including BPH and prostatic neoplasia, or lower urinary tract infection [7], further complicating the diagnostic process [4,8]. Clinical signs are often non-specific and may include lethargy, fever, anorexia, vomiting, urethral discharge and pain on digital rectal palpation, especially when the condition is acute [5,6,9]. Chronic prostatitis is frequently asymptomatic and, when present, its clinical signs often mimic recurrent urinary tract infections [1,7]. Prostatic cysts are frequently observed in prostatic diseases, and in the presence of bacterial infection they may become prostatic abscesses [4,5,6,10]. Given the similarity of clinical signs among different prostatic diseases, a definitive diagnosis often requires a combination of clinical examination, hematological evaluation, diagnostic imaging, and prostatic cytology or culture [5,6]. Dogs with acute bacterial prostatitis typically exhibit hematological evidence of systemic inflammation, including leukocytosis with mature neutrophilia, a left shift, and, in severe cases, a high proportion of neutrophils with toxic granulation, monocytosis, and thrombocytopenia [11].

Ultrasonography is currently considered the imaging modality of choice for the evaluation of the canine prostate because it provides superior soft tissue contrast compared with radiography and is non-invasive, widely available, and suitable for repeated examinations compared with CT [12].

Nevertheless, differentiation among prostatic diseases remains challenging due to overlapping ultrasonographic features [13]. In particular, prostatic enlargement, irregular shape, and parenchymal alterations such as cysts and mineralization may be present in all prostatic conditions, limiting the use of the sole B-mode ultrasound to differentiate these diseases [12,14]. Unlike in dogs, where the prostate is routinely evaluated by transabdominal ultrasonography, transrectal ultrasonography (TRUS) is considered the gold-standard ultrasonographic technique for assessing the human prostate. However, limited availability of suitable probes and the potential need for sedation or anesthesia may have restricted the use of TRUS in dogs. In human medicine, TRUS has identified characteristic features of acute bacterial prostatitis, including prostatic enlargement, heterogeneous echotexture with hypoechoic areas, increased vascularization on Color Doppler imaging, and well-defined hypoechoic fluid collections in cases of prostatic abscesses [15]. Detailed descriptions of ultrasonographic features of prostatitis and prostatic abscesses in dogs are still limited, and the possible associations between imaging findings, clinical presentation, and hematological abnormalities have not been extensively investigated so far [5].

We hypothesized that specific ultrasonographic findings such as prostatic enlargement, size of the abscesses, and inflammatory signs may correlate with clinical and hematological alterations and may therefore contribute to the assessment of disease severity in dogs affected by prostatitis or prostatic abscessation.

The aim of the present study was to describe the ultrasonographic features of canine prostatitis and prostatic abscesses and to investigate their association with clinical presentation and hematological and biochemical findings in order to evaluate the potential diagnostic and prognostic value of ultrasonography in these conditions.

## 2. Materials and Methods

### 2.1. Study Design and Case Selection

The present retrospective observational study included clinical cases of dogs diagnosed with prostatitis, with or without prostatic abscesses. Cases were selected from the medical records of the Clinic of Reproductive Medicine, Vetsuisse Faculty, University of Zurich, using the hospital information system (VETERA®+, Vetera GmbH, Eltville am Rhein, Germany), within the period from 2018 to 2026. The cases were searched using the following keywords: prostatitis, prostatic infection, prostatic inflammation, prostatic abscess and prostatic abscesses.

**Figure 1:**
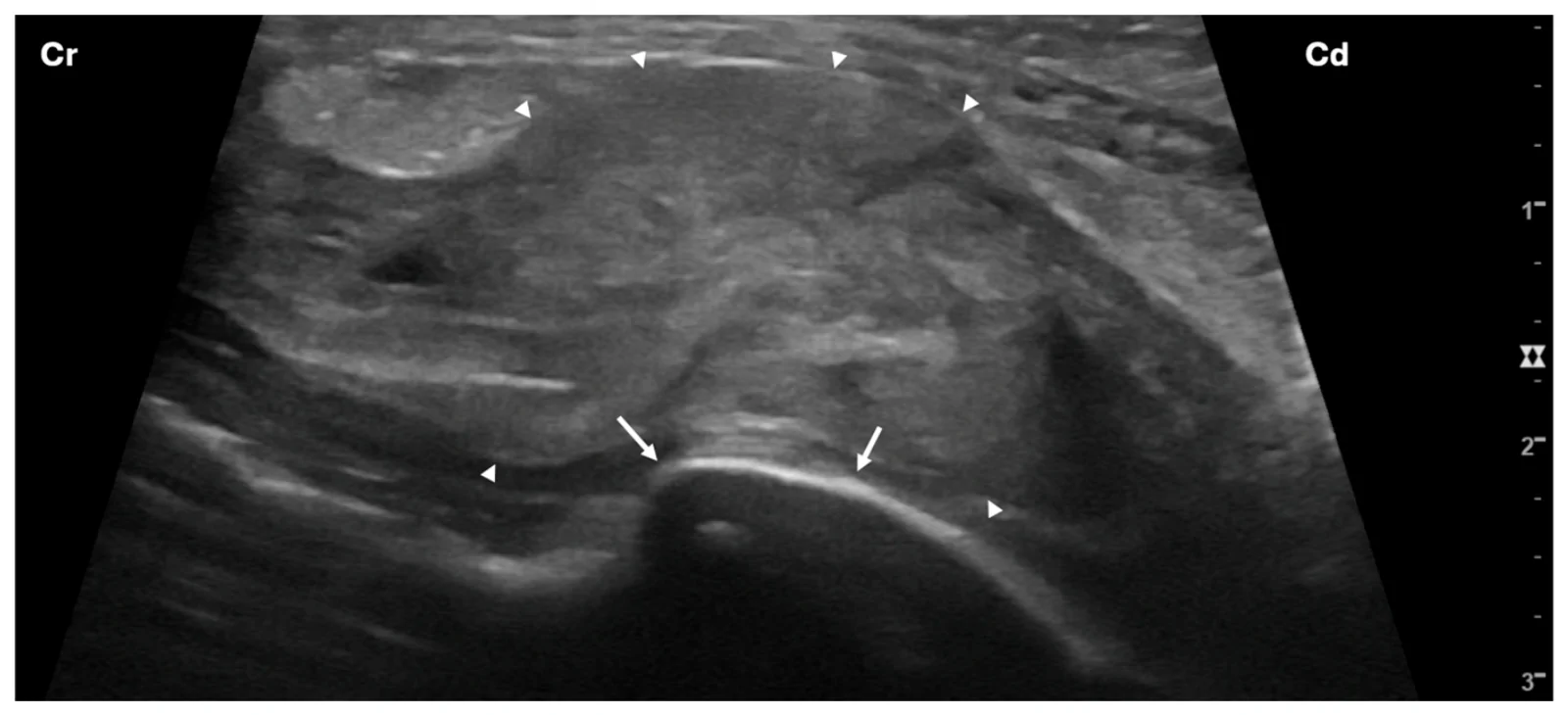
Ultrasound-guided prostatic massage in a 12-year-old intact Bolonka dog. While the catheter is in place (not visible in the picture), a finger (white solid arrows) is introduced into the rectum to perform a massage of the prostate gland (white arrowheads).

Inclusion criteria comprised dogs with a confirmed diagnosis of acute prostatitis based on cytological examination of samples obtained by fine-needle aspiration (FNA) and/or prostatic wash, or on histopathological evaluation of biopsy samples collected during surgery. Cytological diagnosis of prostatitis was based on the clearly visible presence of inflammatory cells, predominantly neutrophils, with or without intracellular bacteria, associated with prostatic epithelial cells and cellular debris, as described by Palmieri et al. 2022 [16]. Most of the prostatic wash procedures consisted of a standardized ultrasound-guided prostatic wash technique adapted from the Meares–Stamey four-glass test commonly used in human andrology [17]. Briefly, after ultrasound-guided urinary catheterization and bladder emptying, urine was collected for urinalysis and culture. The bladder was then flushed with 5–10 mL of sterile saline, which was aspirated and labeled as the pre-prostatic massage sample. The catheter was subsequently withdrawn under ultrasound guidance to the caudal prostatic pole, while a second operator performed digital rectal prostatic massage (Figure 1). During massage, the primary operator repeatedly flushed and aspirated 5–10 mL of sterile saline. Finally, the catheter was repositioned in the urinary bladder and the remaining fluid was collected.

The fluid obtained after prostatic massage was considered the post-prostatic wash fraction and was submitted for cytological and bacteriological evaluation of the prostate gland. All dogs included had signalment data collected (breed, age, body weight, and reproductive status), a complete anamnesis and underwent a complete general physical and andrological examination, hematological and biochemical blood analyses, and ultrasonographic evaluation of the prostate gland. Dogs with incomplete medical records or with only a presumptive diagnosis of prostatitis without cytological or histological confirmation were excluded from the study. Ethical review and approval were not required for this study because it consisted of a retrospective analysis of anonymized clinical data obtained during routine diagnostic procedures. No experimental interventions were performed on client-owned animals. Written owner consent authorizing the use of clinical data for research purposes had been obtained at hospital admission.

### 2.2. Clinical and Ultrasonographic Examination

Clinical examination included a general physical examination and complete andrological evaluation, consisting of examination of the penis and prepuce, inspection and palpation of the scrotum and testicles, and in most cases, digital rectal palpation of the prostate gland or abdomen.

Ultrasonographic examinations were performed by experienced clinicians specialized in small-animal reproductive imaging using an ultrasound machine (LOGIQ S8, GE Healthcare, Wauwatosa, WI, USA) equipped with a microconvex (3–10 MHz) and a linear probe (11 MHz). The operators were not blinded to the anamnesis and clinical findings. Due to the retrospective nature of the study, image settings, including gain, depth, focal zone, and dynamic range, were not standardized and varied among examinations and operators. Ultrasonographic examinations were performed by seven operators routinely involved in the small-animal reproduction clinic, including three ECAR diplomates and four ECAR residents. No interobserver agreement was obtained. All ultrasonographic images were independently reviewed by two experienced operators, and final decisions on the recorded findings were reached by consensus.

B-mode ultrasonographic evaluation of the prostate included assessment of prostatic size, shape, margins, echogenicity, and echotexture. The prostate was measured in two planes: longitudinal and transverse. The length (L), width (W), and height (H) were recorded as the cranio–caudal, latero–lateral, and dorso–ventral measurements, respectively. The actual volume of the prostate (PV) was then calculated using the formula proposed by Ruel et al. (1998) [18]:

PV = (L × W × H) × 0.523,(1)

Additionally, the expected prostatic volume was determined in intact dogs based on their age (A) and body weight (BW), using the formula proposed by Ruel et al. (1998) [18]:

Expected PV = (0.867 × BW) + (1.885 × A) + 15.88,(2)

Ultrasonographic evaluation included assessment of the presence of intraprostatic cystic lesions, mineralization, intracapsular edema, focal or diffuse periprostatic steatitis, peritoneal effusion, and regional lymphadenopathy. Prostatic abscesses were defined as cavitary intraprostatic lesions containing echogenic or hypoechoic fluid or cellular debris, confirmed by at least one of the following: cytology, bacteriology, or surgical exploration. Testicular and urinary bladder ultrasonographic findings were additionally recorded when available. Due to the retrospective nature of the study, imaging protocols and equipment settings were not standardized.

### 2.3. Laboratory and Bacteriological Analysis

Hematological parameters evaluated included hematocrit, platelets, total leukocyte count, band neutrophil count, monocyte count, and the degree of neutrophilic toxicity. Neutrophilic toxicity was also recorded both as a categorical percentage (0, 5–10%, 11–30%, or >30%) and as a semi-quantitative score, used for statistical analysis as appropriate (0 or 1). Serum biochemical parameters included urea, creatinine, bilirubin, alkaline phosphatase activity (ALP), hepatic enzymes (alanine aminotransferase ALT, and aspartate aminotransferase, AST), albumin, glucose, lipase, and C-reactive protein (CRP). Results of bacteriological cultures performed on urine samples and prostatic samples obtained by prostatic wash or FNA of prostatic abscesses were also recorded.

### 2.4. Outcome and Follow-Up

Information regarding the type of treatment (medical or surgical), administered antimicrobial therapy, length of hospitalization, clinical outcome and ultrasonographic findings at follow-up examinations was collected for all dogs.

### 2.5. Statistical Analysis

Statistical analysis was performed using IBM SPSS Statistics (version 30.0; IBM Corp., Armonk, NY, USA). The normality of continuous variables (age, body weight, CRP, hospitalization time, and prostate/abscess dimensions) was assessed using the Shapiro–Wilk test. Continuous variables (CRP, body weight, hospitalization time, prostate size, and abscess diameter) were summarized as median and interquartile range (IQR), and non-parametric tests were used throughout the analysis.

Comparisons between two independent groups (e.g., presence vs. absence of ultrasonographic findings such as edema, free abdominal fluid, steatitis, or mineralization) were performed using the Mann–Whitney U test. Effect size (r) was also calculated.

Associations between categorical variables (e.g., ultrasonographic findings, presence of clinical signs such as inappetence or toxicity) were evaluated using the Chi-square test or Fisher’s exact test when expected cell counts were <5.

Correlations between continuous variables (e.g., CRP, hospitalization time, prostate size, and abscess diameter) and ordinal or continuous predictors were assessed using Spearman’s rank correlation coefficient (rho).

Measured PV was compared with the expected PV calculated according to the published reference formula using the Wilcoxon signed-rank test. Descriptive statistics including median and interquartile range (IQR) were calculated for prostate volume and individual prostatic dimensions (length, width, and height).

The Bonferroni correction was applied to account for multiple comparisons, with corrected significance thresholds calculated for each group of related analyses.

A *p*-value <0.05 was considered statistically significant.

## 3. Results

### 3.1. Animals

A total of 35 male dogs, of which 31 were intact and 4 neutered, with a median age of 9.5 years (IQR = 7.5–11; range 3–13 years) and a median body weight of 27.85 kg (IQR = 15.2–38.5; range 4.66–74 kg) met the inclusion criteria. The dogs recruited represented various breeds including Beauceron (3), Mix Breed (3), French Bulldog (2), Flat Coated Retriever (2), Labrador Retriever (2), Bolonka (2), Yorkshire Terrier (2), Malinois (1), Rottweiler (1), Small Münsterländer (1), Airedale Terrier (1), Boxer (1), Whippet (1), Portuguese Water Dog (1), Australian Kelpie (1), German Shorthaired Pointer (1), American Akita (1), Weimaraner (1), Hungarian Short-Haired Pointer (1), Lhasa Apso (1), Beagle (1), German Shepherd (1), Poodle (1), Small Swiss Hound (1), Dachshund (1) and Field Spaniel (1). Fifty-four dogs were excluded because of other prostatic diseases, while six were excluded because cytological confirmation of prostatitis was unavailable.

Prostatitis was diagnosed cytologically in 14 cases using FNA and in 11 cases using prostatic wash; in the remaining 10 cases, diagnosis was established using both cytology (via FNA or prostatic wash) and histopathology from biopsy samples obtained during surgical treatment of the prostatic disease.

### 3.2. Clinical Examination and Ultrasonographic Findings

The most commonly observed clinical symptoms were fever, apathy, inappetence, defecation abnormalities, lower urinary tract signs, lameness, vomiting, and preputial discharge. Defecation abnormalities included diarrhea, constipation, and tenesmus. Lower urinary tract signs comprised urinary incontinence, haematuria, polyuria, stranguria, and dysuria. Results concerning prostatic pain elicited by attempted palpation were documented in 26 dogs. In 7 cases, a pain reaction was observed during digital rectal examination and in six cases during abdominal palpation. In the remaining 13 dogs with a documented pain assessment attempt, the prostate was not reachable at digital rectal examination in 8 cases and abdominal palpation did not provoke pain reactions, whereas in the other 5 cases, digital rectal examination was feasible, but no pain reaction was detected on either rectal or abdominal palpation.

Except for two dogs presenting urination and defecation disorders for more than 20 days before referral, the interval between onset of clinical signs and clinical presentation was between 1 and 3 days. The frequency of each presenting clinical sign is summarized in Table 1.

Prostatic measurements and their association with body weight are summarized in Table 2. All linear prostatic dimensions showed a moderate positive correlation with body weight (rho = 0.6, *p* < 0.001), while prostatic volume showed a slightly stronger positive correlation (rho = 0.7, *p* < 0.001). The measured prostatic volume did not differ significantly from the expected volume. No significant association was detected between prostatic volume and age.

**Figure 2:**
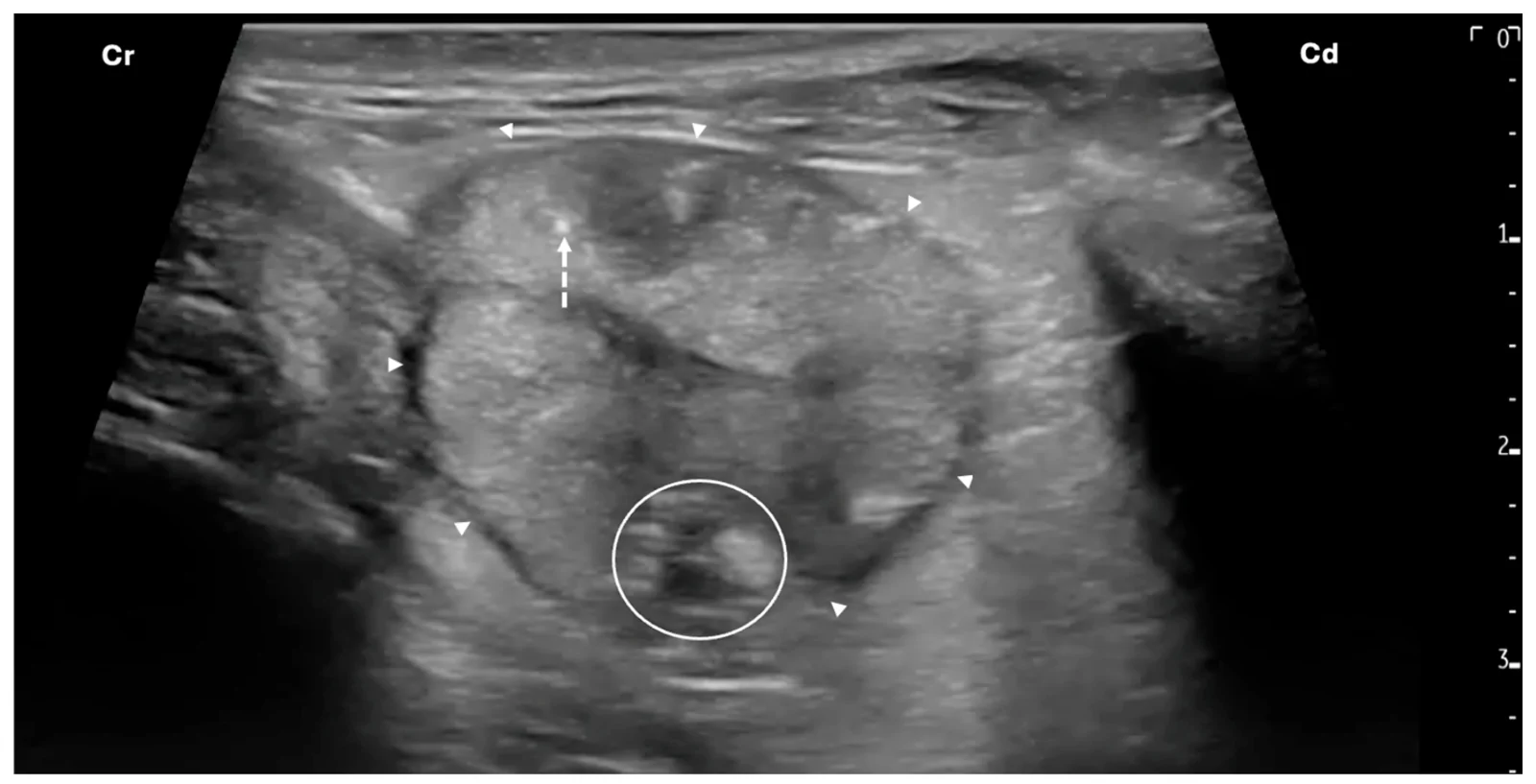
Longitudinal view of the prostate gland affected by prostatitis and with capsular edema in a 10-year-old intact poodle dog. The prostate is surrounded by a hypoechoic band (white arrowheads), suggestive of capsular edema. The parenchyma is inhomogeneous due to the presence of hyperechoic foci suggestive of mineralization (dashed arrows) and cystic lesions (white circle).

**Figure 3:**
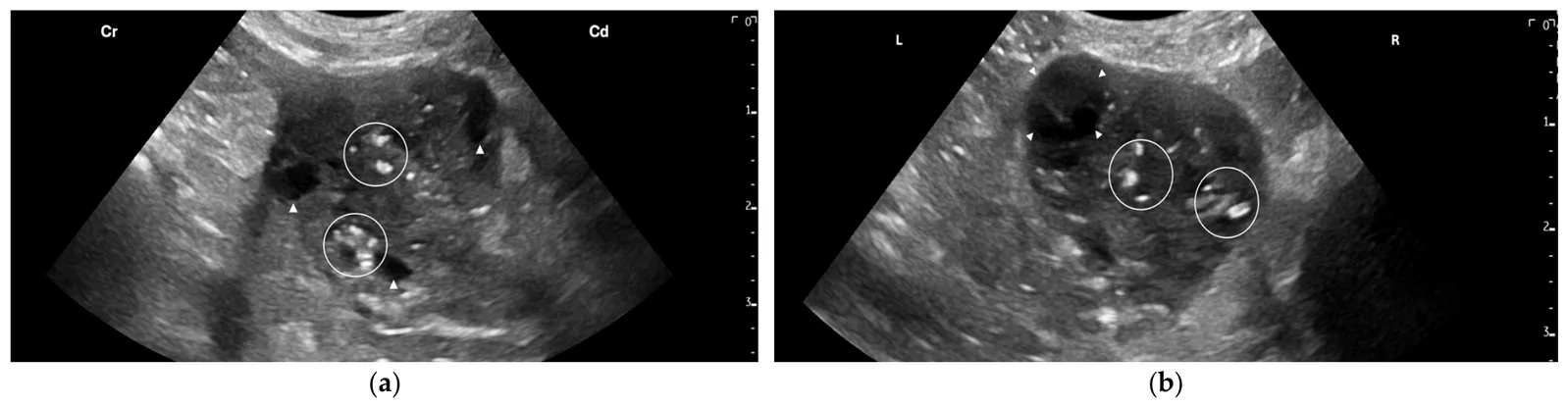
Longitudinal (**a**) and transverse (**b**) ultrasonographic views of the prostate in a 13-year-old intact Lhasa Apso dog with bacterial prostatitis. The prostate is asymmetric due to the presence of several prostatic abscesses (white arrowheads). The prostatic parenchyma is markedly heterogeneous and shows multifocal hyperechoic foci consistent with mineralization (white open circles).

**Figure 4:**
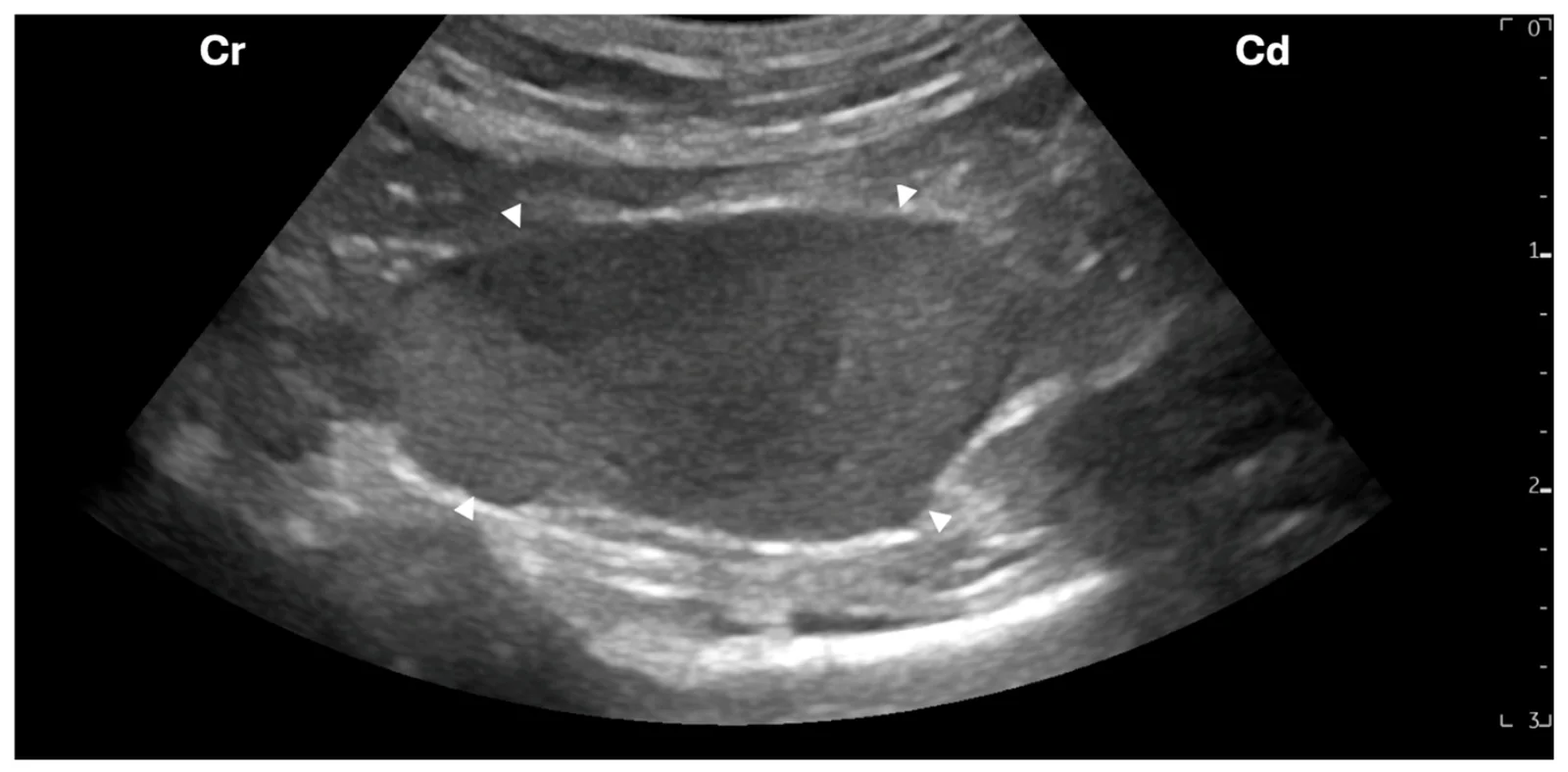
Longitudinal ultrasonographic view of the left medial iliac lymph node (white arrowheads) in a 10-year-old intact mixed-breed dog with acute bacterial prostatitis. The lymph node is enlarged but retains its normal shape, smooth margins, and homogeneous echotexture. No perinodal inflammatory changes are evident.

In all dogs, the prostate appeared enlarged, with irregular margins and inhomogeneous parenchymal echotexture. Small intraparenchymal prostatic cysts ranging from 2 to 6 mm in diameter were detected in all cases. The most frequently observed ultrasonographic findings included capsular edema (Figure 2), periprostatic steatitis/peritonitis, presence of abdominal effusion, intraprostatic parenchymal mineralization (Figure 3) and enlargement of the medial iliac lymph nodes (Figure 4).

**Figure 5:**
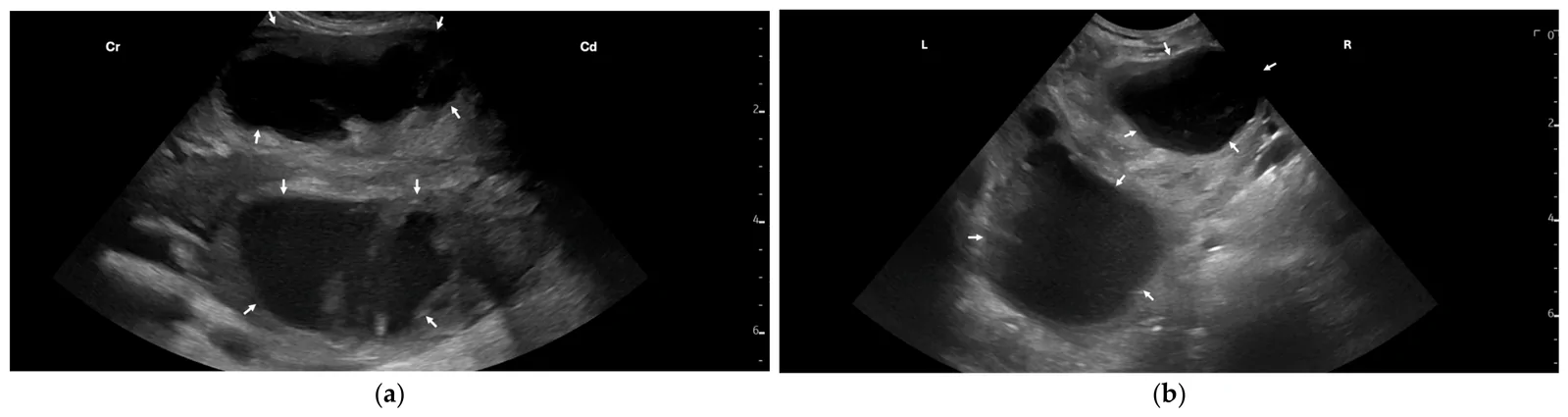
Longitudinal (**a**) and transverse (**b**) ultrasonographic views of the prostate in a 10-year-old intact mixed-breed dog with bacterial prostatitis and multiple prostatic abscesses (white solid arrows). The abscesses occupy most of the prostatic parenchyma, resulting in marked distortion of the normal prostatic architecture and contour.

Intraprostatic abscesses were detected in 25 dogs, whereas in two cases para-prostatic abscesses were observed. The appearance of parenchymal abscesses was variable, most commonly irregular in shape, although some lesions showed a more regular ellipsoid outline (Figure 5).

The location of abscesses within the prostate was variable, with no apparent predilection for a specific region. The number of prostatic abscesses varied among dogs. Four abscesses were identified in two dogs, three abscesses in three dogs, and two abscesses in three dogs. In all remaining cases, only a single abscess was observed. In all cases, abscesses altered the prostatic contour, resulting in asymmetry of the lobes. The content was predominantly hypoechoic with hyperechoic fluctuating foci or heterogeneous echogenic material. The median diameter of the abscesses was 2.4 cm (IQR = 1.3–3.3; range 0.8–6.5 cm). Abscess diameter had a moderately positive correlation with body weight (rho = 0.6; *p* = 0.001), as well as with prostate dimensions, including length (rho = 0.6; *p* = 0.005), width (rho = 0.6; *p* < 0.001), height (rho = 0.6; *p* < 0.001), and PV (rho = 0.6; *p* < 0.01). No correlation was observed between the size of prostatic abscesses and the presence of specific clinical signs. Dogs with a low proportion of neutrophils with toxic granulation in blood had significantly higher mean ranks for abscess diameter compared with dogs with high proportions (*p* = 0.044), although the effect size was small (r = 0.12). This finding was confirmed by Spearman’s rank correlation analysis that revealed a moderate negative correlation between abscess diameter and the proportion of neutrophils with toxic granulation (rho = −0.4, *p* = 0.02). No further correlation was observed between prostatic abscesses and inflammatory blood parameter changes. Ultrasonographic abnormalities of the urinary bladder were common, including features consistent with cystitis. Specifically, altered urine echogenicity (*n* = 26), wall thickening (*n* = 7) and irregular wall outline (*n* = 4), sediment or urolithiasis (*n* = 4) were detected. Testicular abnormalities were mainly represented by neoplastic changes (*n* = 9), of which five were unilateral and four bilateral, or age-related/chronic parenchymal alterations such as smaller size, irregular margins and inhomogeneous echotexture (*n* = 6) [14,19]. The ultrasonographic findings are summarized in Table 3.

Regarding the correlation of clinical signs with ultrasonographic findings, only inappetence was significantly associated with the presence of prostatic edema (χ^2^ test, *p* = 0.024), with a moderate strength of association (Cramer’s V = 0.38), indicating a moderate relationship between inappetence and prostatic edema. Color Doppler ultrasound was performed in just four cases, and settings such as pulse repetition frequency, gain and wall filter were not consistent across examinations; therefore, these data were excluded from the analysis.

### 3.3. Laboratory and Bacteriological Analysis

The most often observed laboratory abnormalities were leukocytosis (*n* = 23) and left shift (*n* = 26). An increased percentage of neutrophils with toxic granulation was observed in 21 cases, with 10 showing 5–10%, 7 showing 11–30% and 4 showing > 30%. In 16 cases, slight anemia was detected. CRP was increased in all dogs with a median level of 150 mg/L (IQR = 24.35–262.95). Increased ALP activity was found in 16 cases.

Bacteriological culture results from the prostate were available for 25 dogs, including 16 dogs in which paired prostatic and urine cultures had been performed. In addition, urine samples from 9 dogs were collected and forwarded for bacteriological examination. Prostatic samples were obtained by ultrasound-guided FNA of the abscess content in 20 cases and by prostatic wash in 5 cases. All urine samples were collected by cystocentesis. The bacterial species isolated from prostatic and urine cultures are summarized in Table 4.

Among the 16 dogs for which both prostatic and urine cultures were available, the same bacterial species was isolated from both samples in 14 cases. In one dog, the urine culture was negative whereas the prostatic culture yielded *Mycoplasma canis*, while in another dog, different bacterial species were isolated from the prostate and urine. Multiple bacterial species were isolated from the prostatic sample in two cases and from the urine sample in three cases. Antibiogram showed similar resistance patterns in both urine and prostate samples, with main resistance occurring against cephalexin and doxycycline. Among the cases with sterile urinary samples, no ultrasonographic bladder abnormalities or just changes in the echogenicity of the urine were detected. In two out of three dogs, in which prostatic culture was sterile, the dog had received antibiotics prior to presentation.

### 3.4. Outcome, Treatment and Follow-Up

Among the 35 dogs included in the study, 31 dogs recovered, whereas four were euthanized because of poor prognosis and/or secondary complications, including sepsis, urethral rupture, oesophageal stricture, and recurrence of abscessation after surgery. One dog was euthanized immediately after diagnosis because of a poor prognosis, while the remaining three dogs were euthanized during the follow-up period.

Treatment included medical management, and additionally fourteen dogs with prostatic abscesses underwent surgical debridement, omentalization, and castration. Surgical omentalization of the abscess was performed as described by White et al. 1995 [20]. In only one dog, intra-cystic administration of an antibiotic was performed after drainage. Two dogs developed urinary incontinence after recovery, both of which had undergone surgical omentalization. The treatment categories were not exclusive, as antimicrobial and androgen deprivation treatment were administered independently of the surgical management of prostatic abscesses.

Medical management included administration of antibiotics, analgesia, androgen deprivation and fluid therapy. All 34 dogs received antimicrobial treatment. In particular, the treatment was initiated intravenously during hospitalization and continued orally after discharge for at least four weeks. A single antimicrobial agent was administered to 24 dogs (68.6%), whereas combination therapy was used in 11 (31.4%). Among dogs treated with a single antimicrobial medication, enrofloxacin was the most frequently prescribed drug (*n* = 17), followed by marbofloxacin (*n* = 3), amoxicillin (*n* = 2), pradofloxacin (*n* = 1), and trimethoprim-sulfamethoxazole (*n* = 1). For dogs receiving combination therapy, the most common regimen was enrofloxacin plus ampicillin (*n* = 9), followed by enrofloxacin plus amoxicillin (*n* = 1) and marbofloxacin plus amoxicillin (*n* = 1).

Concerning androgen deprivation, sixteen dogs underwent surgical castration, while medical treatment consisting of osaterone acetate or finasteride administration was performed in 11 and 3 dogs, respectively. Four dogs did not require additional androgen deprivation because they were already castrated.

Analgesia consisted mainly of opioids, non-steroidal anti-inflammatory drugs, paracetamol and gabapentin.

Hospitalization time was a median of 3 days (IQR = 1–6). Dogs with inappetence (*p* = 0.007; r = 0.43), defecation problems (*p* = 0.033; r = 0.35) and increased toxic granulation values according to blood results (*p* = 0.023; r = 0.5) also required longer hospitalization.

**Figure 6:**
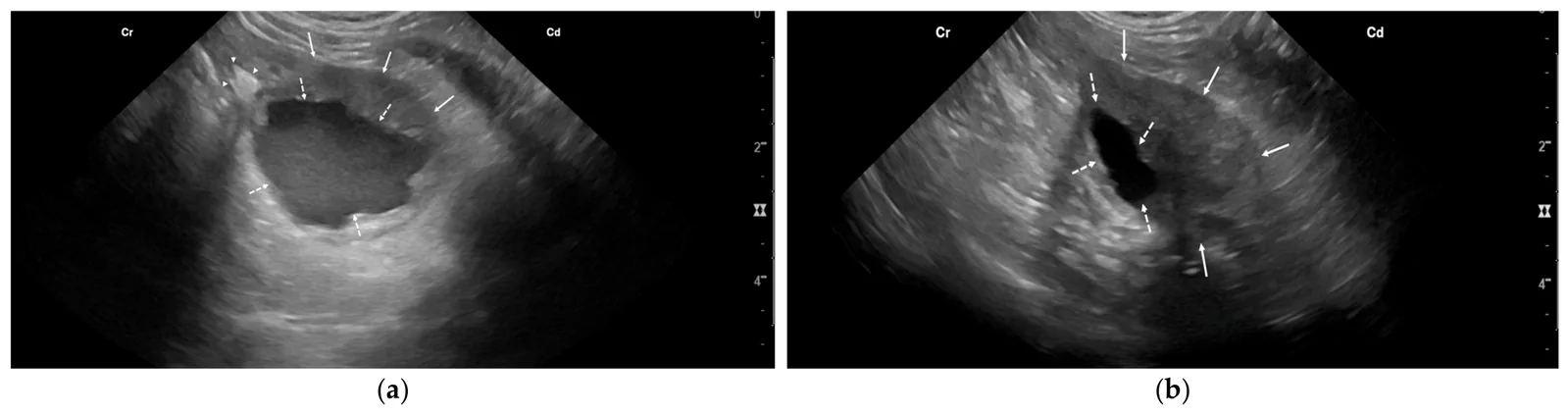
Prostatic abscess in an 8-year-old castrated Field Spaniel dog pre (**a**) and post (**b**) medical treatment. (**a**) The prostate gland (white arrows) is characterized by the presence of a large prostatic abscess (white dashed arrows) with irregular shape and contour and echogenic fluid content. The fat surrounding the prostate gland appears hyperechoic, being suggestive of steatitis (white arrowheads). (**b**) After one month of antimicrobial treatment, the abscess appears smaller in size, still irregular in shape, but with clear anechoic fluid, and the fat steatitis was no longer present.

Twenty-four dogs underwent a standardized follow-up examination one month after first presentation, corresponding to the end of the planned duration of antibiotic treatment. Although four dogs were examined also before or after this time point, these examinations were not standardized in terms of time planning and were therefore excluded from the follow-up analysis. One month after presentation, the prostate decreased in size in all dogs but retained an inhomogeneous echotexture due to the presence of hyperechoic parenchymal stippling. Moreover, a reduction in abscess size was observed, accompanied by a shift in the fluid content to an anechoic appearance (Figure 6).

In dogs undergoing surgical debridement and omentalization, the omentum was visible within the prostate gland and was characterized by an increased echogenicity. Edema of the capsule and abdominal effusion resolved in most cases after one month. Periprostatic steatitis persisted in three cases but subjectively improved compared with previous examinations. Abscesses recurred in two dogs that underwent surgical treatment. In one of these two cases, a cyst showed communication with the urethra and the dog was euthanized, whilst the other one underwent a new surgical treatment that solved the condition, as confirmed by clinical examination and ultrasound.

## 4. Discussion

To the authors’ knowledge, this is one of the largest case series specifically characterizing the ultrasonographic appearance of canine prostatitis and prostatic abscess. Although prostatitis represents the second most common prostatic disorder in dogs [6], its ultrasonographic appearance has received limited attention in the veterinary literature [5,6,14,21].

The most common ultrasonographic findings observed in the assessed cases were prostatic enlargement, irregular margins, inhomogeneous parenchymal echotexture, asymmetry of the prostatic lobes, capsular edema, and periprostatic steatitis.

Interestingly, prostatic dimensions and volume were positively correlated with body weight but not with age, which may be attributable to the fact that the included dogs were all relatively old and therefore exhibited limited age-related variation. Furthermore, measured prostatic volume did not differ significantly from expected values calculated using the formula proposed by Ruel et al. [18]. This finding may reflect that the reference equation already accounts, at least partially, for age-related prostatic enlargement in the general canine population. Therefore, the “expected” volume may not represent a normal prostate but rather a population-based reference value that includes already age-related changes. This interpretation is further supported by the findings of Posastiuc et al., who reported a volume ratio (measured/expected prostatic volume) of 1.29 in dogs with BPH and concurrent prostatitis. Although this indicated an increased prostatic volume relative to the expected value, the ratio did not differ significantly from that reported in dogs with subclinical BPH (1.01) or clinical BPH (1.13) [22]. These findings suggest that the Ruel formula may already account for a proportion of the age-related increase in prostatic volume. The prostatic volume and individual dimensions observed in the present study were also comparable with those previously reported in dogs with BPH [23,24], and the prostate size appeared increased compared with values reported for healthy dogs in previous studies [18,23,25]. Moreover, the condition was diagnosed as acute based on cytology and clinical signs onset. However, as no previous history concerning prostate health was available, it was not possible to assess how long the condition had been present and whether chronic changes such as fibrosis may have already developed. Therefore, based on our findings, prostatitis appeared to affect prostatic morphology and architecture more than total gland volume. In many cases, inflammatory lesions and abscesses resulted in lobar asymmetry, irregular margins, and marked parenchymal heterogeneity without substantial changes in overall size. These findings suggest that volumetric assessment alone has limited diagnostic value for prostatitis and should always be interpreted in conjunction with other qualitative ultrasonographic features.

Alterations in echotexture represent one of the most consistent indicators of prostatic disease, with cystic changes, fibrosis, inflammatory infiltrates, or focal mineralization being the most common findings. Consequently, inhomogeneous echotexture should be considered a sensitive but non-specific marker of prostatic pathology, as stated by other authors [12,22].

Small intraparenchymal cystic lesions were identified in almost all dogs included in the study, in agreement with previous reports [21]. Given that most dogs were intact and belonged to an age group commonly affected by BPH, these changes may reflect concurrent hyperplastic disease. However, cysts may also occur secondary to chronic inflammation and ductal obstruction; therefore, the exact relationship between cyst and prostatitis remains unclear and deserves further investigation.

Intraprostatic mineralization was observed in fewer than half of the cases, in agreement with previous studies [5]. Mineralization is a non-specific finding reported in BPH, chronic prostatitis, and neoplasia. From a pathological perspective, these deposits most likely represent dystrophic mineralization secondary to chronic inflammation, tissue degeneration, or necrosis. Since similar necrotic processes may occur in both inflammatory and neoplastic conditions, mineralization alone cannot be considered diagnostically specific [16]. Bradbury et al. (2009) reported a high positive predictive value of mineralization for prostatic neoplasia in neutered dogs [13]. However, this association appeared less reliable in intact dogs. The limited number of castrated dogs in the present study did not allow comparison.

Abscess size was highly variable and showed a moderate positive correlation with body weight and prostatic dimensions. The minimum size of prostatic abscesses detectable by ultrasonography could not be established because of the retrospective nature of the study. Although the smallest abscess identified in the present population measured 0.8 cm and was confirmed by cytology, smaller cavitary lesions with purulent content may have been present but could not be definitively classified as abscesses in the absence of confirmatory diagnostic findings. Interestingly, larger abscesses were associated with lower proportions of neutrophils with toxic granulation. One potential explanation is that larger lesions may represent more chronic and compartmentalized abscesses, in which the inflammatory process becomes locally contained, whereas smaller lesions may correspond to more acute stages of infection associated with a stronger systemic inflammatory response. Alternatively, differences in host immune response or bacterial virulence may have contributed to this observation. Given the limited sample size, this finding should be interpreted cautiously, and further studies are required to clarify the relationship between abscess maturation and systemic inflammatory markers. Abscess morphology was also variable, although irregular shapes were more frequently observed than well-defined spheroidal lesions. The internal content was consistently hypoechoic to echogenic and often contained suspended echogenic material, rather than being purely anechoic. Follow-up examinations demonstrated progressive changes in echogenicity during treatment, with lesions becoming more anechoic over time. This may reflect a reduction in cellularity and inflammatory debris, suggesting that ultrasonographic appearance could potentially be used as an indirect marker of lesion evolution. However, further prospective studies correlating ultrasonographic findings with cytological and bacteriological results are required before definitive diagnostic criteria can be established.

Capsular edema, steatitis, and abdominal effusion were among the most common ultrasonographic findings. These changes have previously been described in inflammatory prostatic disease [14]. As these findings are not typically associated with BPH, they may aid in the differential diagnosis. However, prostatic neoplasia may have a highly variable presentation, often showing signs of inflammation that may be confused with prostatitis without underlying neoplasia [10]. Neoplastic growth may determine changes in the parenchymal architecture, like the presence of big cysts or necrosis [10,16,26], which may favor the growth of bacteria, determining the development of prostatitis as a complication. Given the overlap in clinical signs and imaging findings among prostatic diseases, cytological evaluation is still recommended. However, caution should be exercised when performing FNA if prostatic carcinoma is also suspected in the differential diagnosis because of the potential risk of tumor seeding [1,27]. In such cases, prostatic wash cytology and bacteriological culture may represent a suitable first-line diagnostic approach, as they may reduce the risk of tumor seeding while still providing valuable diagnostic information. Further studies should be performed to compare the prostatic sampling methodologies.

Medial iliac lymph node enlargement has been reported in association with reproductive tract disease [28,29,30]. In the present study, medial iliac lympho-adenomegaly was reported in 20 cases, suggesting a potential sign of inflammation; however, it was not associated with specific clinical signs or prognosis. Their involvement has also been described in cases of prostatic neoplasia with metastasis [10,26]; therefore, their examination should be included in the ultrasonographic assessment of the male reproductive tract.

Bladder abnormalities, mainly consistent with cystitis, were commonly observed in the present study. This finding is not surprising considering the close anatomical and functional relationship between the urinary and reproductive tracts. Ascending bacterial infections may spread from the lower urinary tract to the prostate gland, while prostatic infections may also contribute to urinary tract inflammation [7]. Nevertheless, the presence of infection in one compartment does not necessarily imply involvement of the other. Lea et al. (2022) reported concordance between urine and prostatic bacterial cultures in only 50% of dogs with prostatitis, and in 71% of cases with positive prostatic cultures, urine cultures were negative [5]. Few discordant bacteriological findings between urine and prostatic samples were also observed in the present study, further supporting the concept that urine culture alone may not accurately reflect the microbial status of the prostate gland. Therefore, direct sampling of the prostate, either through prostatic wash or drainage of the abscesses, should be considered whenever prostatitis is clinically suspected [1,9,31]. The frequent detection of bladder wall thickening, irregular wall margins, and altered urine echogenicity may indicate concurrent lower urinary tract inflammation. However, in some dogs with sterile urinary tract findings, the only ultrasonographic abnormality consisted of increased echogenicity of the urine without associated bladder wall changes. Echogenic urinary content alone should be interpreted cautiously. Abnormal debris can be caused by a technical artifact or a pathologic condition such as hematuria, pyuria, crystalline matrix, fat droplet, cellular debris, and inflammatory products [32,33,34]. Consequently, ultrasonographic findings should always be interpreted in conjunction with clinical, bacteriological and cytological results.

Concerning clinical examination, digital rectal palpation is regarded to be an important technique for the examination and diagnosis of prostatitis [1,9]. Abdominal pain and pain upon digital rectal examination may serve as additional indicators of prostatic inflammation and were observed in 13 out of the 26 cases in which pain response was assessed. According to the medical records, no pain response could be elicited upon digital rectal palpation in five cases. In a further eight cases, the prostate could not be reached during digital rectal examination, which is consistent with previous reports describing cranial displacement of the prostate in dogs with BPH [29]. In these dogs, abdominal palpation likewise did not reveal signs of pain, although it remains unclear whether prostatic manipulation was sufficiently intensive to reliably assess pain response. Pain assessment was not performed or documented in nine cases, despite prostatic tenderness being considered a classical feature of prostatic inflammation in veterinary textbooks. This may reflect a reduced emphasis on pain assessment in contemporary clinical practice, where advanced imaging and complementary diagnostic tests frequently provide sufficient evidence to establish the diagnosis.

Most cases recovered with an overall good prognosis after medical or combined medical–surgical treatment. However, possible complications may occur in the future and need to be considered on a case-by-case basis. Recurrence was observed in a small number of cases that underwent surgical management, consistent with previous reports [5,20]. An interesting finding of the present study was the ultrasonographic evolution of prostatic lesions during follow-up examinations. One month after treatment initiation, a reduction in prostate size was observed in all dogs, including the fourteen treated medically with osaterone acetate or finasteride and the remaining dogs that underwent surgical castration. Inflammatory changes such as capsular edema and abdominal effusion had completely resolved. Nevertheless, periprostatic steatitis may persist longer, as found within the present study. The reduction in prostatic size may be related to therapies aimed at androgen suppression, including osaterone acetate or finasteride administration or surgical castration [35,36,37]. Moreover, it is unclear whether prostate regression occurs similarly whether an inflammatory process is present or not. Although in a rat model finasteride significantly reduced bacterial load and inflammatory cell infiltration in prostatic tissue compared with controls [38], there is currently no evidence that androgen deprivation directly improves treatment outcomes or accelerates the resolution of prostatitis in dogs, even though several studies suggest this treatment approach [1,4,9].

The absence or reduction of capsular edema, periprostatic steatitis, and abdominal effusion in most cases one month after treatment initiation suggests that these ultrasonographic abnormalities are primarily associated with active inflammation and may therefore represent useful markers for monitoring therapeutic response. In contrast, the prostatic parenchyma frequently remained heterogeneous despite clinical recovery, mainly due to persistent hyperechoic stippling and architectural distortion. This finding suggests that complete ultrasonographic normalization does not necessarily occur immediately after resolution of infection, and that residual parenchymal alterations likely reflect fibrosis, tissue remodeling, or chronic structural changes secondary to inflammation, as described in chronic prostatitis [14].

Similarly, prostatic abscesses decreased in size following treatment, and their contents progressively became more anechoic. Serial ultrasonographic assessment of abscesses may provide useful information on lesion evolution and treatment response.

Interestingly, dogs presenting with inappetence, defecation abnormalities, or a higher proportion of neutrophils with toxic granulation required significantly longer hospitalization periods. These findings suggest that systemic clinical signs and markers of systemic inflammation may better reflect disease severity and short-term management requirements than ultrasonographic features of the prostate alone. However, although all dogs underwent a complete clinical and ultrasonographic examination, the retrospective nature of the study does not allow complete exclusion of concurrent subclinical diseases that may have influenced some clinical or hematological findings.

The present study has several limitations, including its retrospective design, the relatively small number of cases, and the lack of standardized ultrasonographic protocols due to the involvement of multiple operators. Furthermore, follow-up data and bacteriological results were not available for all dogs. However, recent guidelines for bacterial infection of the urogenital tract suggest placing greater emphasis on clinical signs and ultrasonographic improvement, rather than repeated testing, when assessing treatment response [39].

Because only cytologically or histologically confirmed cases were included, the study population may have been biased toward dogs with more severe disease and may not completely represent the spectrum of prostatitis encountered in general practice. Therefore, the present findings should be confirmed in prospective studies. Follow-up data were not available for all dogs, which limited the assessment of long-term treatment response and prognostic outcome. However, the one-month follow-up was standardized among dogs with available follow-up data, whereas examinations performed at other time points were excluded because their timing was not consistent across the study population. Another limitation is the lack of information on the dogs’ pre-existing prostatic health status, which precludes determining whether some ultrasonographic changes were already present before the inflammatory process or developed as a consequence of it. Moreover, the retrospective design introduced potential bias related to the standardization of ultrasonographic examinations, as they were performed by different operators and a standardized scanning protocol was not prospectively applied. This may have introduced variability in image acquisition, measurements, and assessment of ultrasonographic findings.

B-mode ultrasound represents only one of the ultrasonographic techniques available and is generally considered the first-level imaging modality for the assessment of the prostate. Other ultrasonographic techniques such as Color Doppler and contrast-enhanced ultrasound have been investigated, with some studies showing that a multiparametric ultrasound approach may improve diagnostic differentiation of prostatic conditions [12]. However, further standardization of imaging techniques, acquisition protocols, and interpretation of the results is still required to facilitate their implementation in routine clinical practice.

## 5. Conclusions

This study provides a detailed description of the ultrasonographic features of canine prostatitis and prostatic abscessation, highlighting their variability and frequent overlap with other prostatic disorders. B-mode ultrasonography appears to be a valuable tool for detecting prostatic abnormalities and monitoring treatment response, especially in cases complicated by abscess formation. In particular, capsular edema, abdominal fluid effusion, periprostatic steatitis and medial iliac lymph node enlargement were the main findings associated with prostatitis.

However, many findings remain non-specific, underscoring the need for a multimodal diagnostic approach integrating imaging, clinical assessment, and laboratory data. Future prospective studies are warranted to better define the prognostic value of specific ultrasonographic features and to further clarify their relationship with systemic inflammatory markers and disease chronicity.

## Figures and Tables

**Table 1:** Observed clinical signs in 35 dogs diagnosed with acute prostatitis.

Clinical Signs Observed	Number of Cases	Percentage (%)
Apathy/lethargy	29	82.7
Defecation abnormalities	23	65.7
Inappetence	19	54.3
Vomiting	15	42.9
Pain at abdominal or digital rectal palpation	13	37.1
Lower urinary tract signs	11	31.4
Fever	10	28.4
Lameness	5	14.3
Preputial discharge	5	14.3

**Table 2:** Ultrasonographic prostatic measurements and their correlation with body weight and age in 35 dogs diagnosed with acute prostatitis.

Prostatic Measurements			Body Weight	Age
Variable	Median	IQR	Correlation with BW	Correlation with Age
Length (cm)	5.4	4.4–6.4	Rho = 0.6, *p* < 0.001	Rho = −0.1, *p* = 0.52
Width (cm)	4.4	3.4–5.5	Rho = 0.6, *p* < 0.001	Rho = −0.3, *p* = 0.13
Height (cm)	4.3	2.8–5.1	Rho = 0.6, *p* < 0.001	Rho = −0.001, *p* = 0.99
PV (cm^3^)	51.19	27.96–81.08	Rho = 0.7, *p*< 0.001	Rho = −0.1, *p* = 0.61
Expected PV (cm^3^)	56.39	48.34–64.11		

**Table 3:** Ultrasonographic findings in 35 dogs diagnosed with acute prostatitis.

Ultrasonographic Findings	Number of Cases	Percentage (%)
Steatitis	32	91.4
Bladder abnormalities	26	74.3
Capsular edema	23	65.7
Reactivity of medial iliac lymph nodes	20	57.1
Abdominal effusion	18	51.4
Testicular abnormalities	15	42.9
Mineralization	8	22.8

**Table 4:** Bacterial species isolated from both urine and prostatic samples from 35 dogs with acute prostatitis.

Bacterial Isolates	Urine (*n* = 25)	Prostatic Sample (*n* = 25)
*Escherichia coli*	15	15
*Staphylococcus pseudointermedius*	3	2
*Pseudomonas aeruginosa*	1	1
*Mycoplasma canis*	1	2
*Staphylococcus epidermidis*	2	2
*Streptococcus canis*	1	2
*No bacteria isolated*	7	3

## Data Availability

The raw data supporting the conclusions of this article will be made available by the authors on request.

## Abbreviations

The following abbreviations are used in this manuscript:

BPH	Benign prostatic hyperplasia
TRUS	Transrectal ultrasound
FNA	Fine-needle aspiration
PV	Prostate volume
L	Length
H	Height
W	Width
CRP	C-reactive protein
ALT	Alanine aminotransferase
AST	Aspartate aminotransferase
ALP	Alkaline phosphatase
